# Implications of Programmed Death Ligand-1 Positivity in Non-Clear Cell Renal Cell Carcinoma

**DOI:** 10.15586/jkcvhl.2018.107

**Published:** 2018-10-13

**Authors:** Juan Chipollini, Mounsif Azizi, Charles C. Peyton, Dominic H. Tang, Jasreman Dhillon, Philippe E. Spiess

**Affiliations:** 1 Department of Genitourinary Oncology, H. Lee Moffitt Cancer Center and Research Institute, Tampa, FL, USA; 2 Department of Anatomic Pathology, H. Lee Moffitt Cancer Center and Research Institute, Tampa, FL, USA

**Keywords:** non-clear cell renal cell carcinoma, prognosis, programmed death ligand-1, renal cell cancer, tumor marker

## Abstract

The purpose of this study was to assess the prognostic value of programmed death ligand-1 (PD-L1) positivity in a non-clear cell renal cell carcinoma (non-ccRCC) cohort. PD-L1 expression was evaluated by immunohistochemistry (IHC) using formalin-fixed paraffin-embedded (FFPE) specimens from 45 non-ccRCC patients with available tissue. PD-L1 positivity was defined as ≥1% of staining. Histopathological characteristics and oncological outcomes were correlated to PD-L1 expression. Cancer-specific survival (CSS) and recurrence-free survival (RFS) stratified by PD-L1 status were estimated using the Kaplan–Meier method. Median age was 58 years and median follow-up was 40 months. Non-ccRCC subtypes included sarcomatoid (*n* = 9), rhabdoid (*n* = 6), medullary (*n* = 2), Xp11.2 translocation (*n* = 2), collecting duct (*n* = 1), papillary type I (*n* = 11), and papillary type II (*n* = 14). PD-L1 positivity was noted in nine (20%) patients. PD-L1 positivity was significantly associated with higher Fuhrman nuclear grade (P = 0.048) and perineural invasion (P = 0.043). Five-year CSS was 73.2 and 83% for PD-L1 positive and negative tumors, respectively (P = 0.47). Five-year RFS was 55.6 and 61.5% for PD-L1 positive and negative tumors, respectively (P = 0.58). PD-L1 was expressed in a fifth of non-ccRCC cases and was associated with adverse histopathologic features. Expression of biomarkers such PD-L1 may help better risk-stratify non-ccRCC patients to guide treatment decisions and follow-up strategies.

## Introduction

Renal cell carcinoma (RCC) is a lethal urologic malignancy accounting for 62,700 new cases and 14,240 deaths per year in the Unites States (1). Although diagnosis in earlier stages has become more frequent due to increased use of abdominal imaging, mortality rates have not decreased (2, 3). In cases of clinically localized disease, surgical resection remains the mainstay of curative treatment although recurrences can occur in 20–40% of cases (4).

RCC is considered to be immuno-responsive with cases of complete regression reported with high-dose immunotherapy (5). Newer treatments have mainly relied on the vascular endothelial growth factor (VEGF) or mammalian target of rapamycin (mTOR) pathways, although no durable responses have been observed to date (6, 7). Recent advances in the understanding of molecular contributors of RCC have led to the emergence of novel therapeutic agents such as immune checkpoint inhibitors (8). In 2015, Nivolumab, an anti-programmed cell death-1 (PD-1) inhibitor, was approved for patients with advanced clear cell RCC (ccRCC) with failed prior anti-angiogenic therapy due to significantly higher objective response rates and an acceptable toxicity profile (9, 10).

Despite exciting breakthroughs and increased knowledge of the immune response in RCC carcinogenesis, a significant proportion of patients with non-clear cell RCC (non-ccRCC) subtypes were excluded from pivotal clinical trials. This, in turn, limits the available data needed for the development of evidence-based recommendations. Thus far, the prognostic value of programmed death ligand-1 (PD-L1) positivity in non-ccRCC remains unclear. In this study, we sought to examine the clinical significance of PD-L1 expression in a contemporary cohort of patients treated for non-ccRCC at a single institution.

## Materials and Methods

### Patient selection

A cohort of non-ccRCC patients treated between 2005 and 2015 was retrospectively identified from our RCC database. Tissue was available for 45 patients with non-clear cell subtypes. The study was approved by the institutional review board.

### PD-L1 Immunohistochemistry (IHC)

Tissue microarray (TMA) blocks were prepared from 45 non-ccRCC formalin-fixed paraffin-embedded (FFPE) specimens. A hematoxylin and eosin (H&E) slide of each archival specimen was evaluated, and a block representing the overall tumor was chosen for TMA preparation. Three cores of 1-mm diameter per case were selected and sections were cut into 4-μm thicknesses and placed on positively charged slides for immunostaining. For cases with sarcomatoid or rhabdoid features, only the non-clear cell component was evaluated. Slides were subjected to immunostaining using the Ventana Discovery XT automated system (Ventana Medical Systems, Tucson, AZ) using commercially available antibodies against PD-L1 (E1L3N; Cell Signaling Technology, Beverly, MA). PD-L1 expression was defined in tumor cells if membranous alone or membranous and cytoplasmic staining was present. The positivity was further subclassified as ≥1, ≥5, ≥10, ≥25, and ≥50%. PD-L1 positivity of tumor cells was defined as ≥1% of staining. This cutoff level was based on published data showing successful anti-PD-1 therapy in patients with low PD-L1 expression (11). An expert in genitourinary pathology (J.D.) completed this work and decided on IHC positivity.

There is no definitive cutoff for PD-L1 positivity, with more than one threshold being reported for most antibodies in the literature. Published data for PD-L1 tests are mainly focused on immunohistochemistry tests for lung cancer. The variability in test cutoffs and standards for PD-L1 testing suggests that there is presently no standardized approach. Data on renal cell cancers are scarce. Hence, in this study we followed the approach that has been followed by many published studies in the literature based on lung cancer where the cutoff value of ≥1% for PDL-1 has been considered positive. According to Festino et al., there is no definitive threshold result that can be universally applied to predict clinical response to PD-L1–targeted precision treatments (12).

### Statistical analysis

The association between PD-L1 expression status and histopathologic characteristics was evaluated using the Mann–Whitney or chi-square test and Fisher’s exact test for numeric and categorical variables, respectively. Data were expressed as median and interquartile range (IQR) for continuous variables, and binary variables were reported as counts and percentages (%). Survival endpoints evaluated were cancer-specific survival (CSS) and recurrence-free survival (RFS) and were defined as the time from surgery to death from disease and recurrence of disease, respectively. CSS and RFS stratified by PD-L1 status were estimated using the Kaplan–Meier method. Statistical significance was defined as P < 0.05. All statistical analyses were performed using Statistical Package for the Social Sciences (SPSS), version 24.0 (IMB Corp, Armonk, NY).

## Results

### Clinicopathologic characteristics

Between 2005 and 2015, we identified 45 non-ccRCC patients with available tissue. Clinicopathologic characteristics are provided in Table 1. Median age was 58 years (IQR: 53–68.5). Of the patients, 13 (29%) underwent partial nephrectomy and 32 (71%) underwent radical nephrectomy. Regional lymph node dissection was performed in seven (16%) patients, while inferior vena cava thrombectomy was completed in three (6.7%) patients. Histology subtypes included sarcomatoid RCC (*n* = 9), rhabdoid RCC (*n* = 6), medullary RCC (*n* = 2), Xp11.2 translocation RCC (*n* = 2), collecting duct RCC (*n* = 1), papillary type I RCC (*n* = 11), and papillary type II RCC (*n* = 14). Median tumor size was 7 cm (IQR: 4.5–12.3). Approximately 51% of tumors were pT3–4 and 75.5% were Fuhrman nuclear grades 3–4.

**Table 1:** Patients and tumor characteristics.

*Patients characteristics (n = 45)*	
Median age, years (IQR)	58 (53–68.5)
Gender (%)	
Male	34 (75.6)
Female	11 (24.4)
Race (%)	
White	27 (60)
Non-white	18 (40)
Karnofsky Performance Scale (%)	
<80%	8 (17.8)
≥80%	37 (82.2)
Surgical procedure (%)	
Partial nephrectomy	13 (28.9)
Radical nephrectomy	32 (71.1)
Regional lymph node dissection	7 (15.6)
IVC thrombectomy	3 (6.7)
*Tumor characteristics (n = 45)*	
Non-clear cell subtypes (%)	
Sarcomatoid RCC	9 (20)
Rhabdoid RCC	6 (13.3)
Medullary RCC	2 (4.4)
Xp11.2 translocation RCC	2 (4.4)
Collecting duct RCC	1 (2.2)
Papillary type 1 RCC	14 (31.1)
Papillary type 2 RCC	11 (24.4)
AJCC pathologic T stage (%)	
pT1	16 (35.6)
pT2	6 (13.3)
pT3	21 (46.7)
pT4	2 (4.4)
AJCC clinical M stage (%)	
cM0	39 (86.7)
cM1	6 (13.3)
Median tumor size, cm (IQR)	7 (4.5–12.3)
Fuhrman nuclear grade (%)	
I	3 (6.7)
II	8 (17.8)
III	19 (42.2)
IV	15 (33.3)
Adverse features (%)	
Lymphovascular invasion	12 (26.7)
Tumor necrosis	23 (51.1)
Perineural invasion	1 (2.2)
Tumor thrombus	8 (17.8)
Surgical margin status	
Negative	40 (88.9)
Positive	4 (8.9)
Unknown	1 (2.2)
PD-L1 expression status	
<1% (negative)	36 (80)
≥1% (positive)	9 (20)

### PD-L1 expression and histopathologic characteristics in non-ccRCC

**Figure 1:**
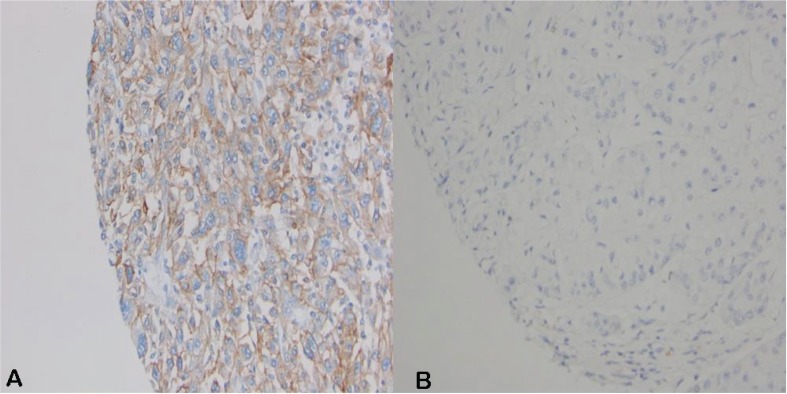
FFPE non-ccRCC specimens stained with anti-PD-L1 antibody (E1L3N). (A) Positive PD-L1 staining; ≥50% positivity (20X). (B) Negative PD-L1 staining; <1% positivity (20X).

PD-L1 positivity on tumor cells was noted in nine (20%) patients (Figure 1), including two papillary type 2 (18%), three sarcomatoid (33%), three rhabdoid (50%), and one Xp11.2 translocation tumors (50%). PD-L1 positivity was significantly associated with higher Fuhrman nuclear grade (P = 0.048) and perineural invasion (P = 0.043) (Table 2). Although not statistically significant, patients with PD-L1-positive tumors had higher pT stage, more cM1 disease, greater tumor size, more lymphovascular invasion, and more tumor necrosis.

**Table 2:** Association of PD-L1 expression and histopathologic characteristics in non-ccRCC.

Characteristic	PD-L1(-) *n* = 36	PD-L1(+) *n* = 9	P value
AJCC pathologic (pT) (%)			
pT1/2	19 (52.8)	3 (33.3)	0.252
pT3/4	17 (47.2)	6 (66.7)	
AJCC clinical (cM) (%)			
cM0	32 (88.9)	7 (77.8)	0.344
cM1	4 (11.1)	2 (22.2)	
Median tumor size, cm (IQR)	7 (4.5–12.6)	8.5 (3.8–12.2)	0.865
Fuhrman nuclear grade (%)			
I/II	9 (25)	2 (22.2)	**0.048**
III/IV	27 (75)	7 (77.8)	
Lymphovascular invasion (%)			
Negative	28 (77.8)	5 (55.6)	0.225
Positive	8 (22.2)	4 (44.4)	
Perineural invasion (%)			
Negative	36 (100)	8 (88.9)	**0.043**
Positive	0 (0)	1 (11.1)	
Tumor necrosis (%)			
Negative	18 (50)	3 (33.3)	0.303
Positive	18 (50)	6 (66.7)	
No. Non-clear cell subtypes (%)			
Sarcomatoid RCC	6 (66.6)	3 (33.3)	0.135
Rhabdoid RCC	3 (50)	3 (50)	
Medullary RCC	2 (100)	0 (0)	
Xp11.2 translocation RCC	1 (50)	1 (50)	
Collecting duct RCC	1 (100)	0 (0)	
Papillary type 1 RCC	14 (100)	0 (0)	
Papillary type 2 RCC	9 (81.8)	2 (18.2)	

Bold values indicate p<0.05

### PD-L1 expression and oncological outcomes in non-ccRCC

**Figure 2:**
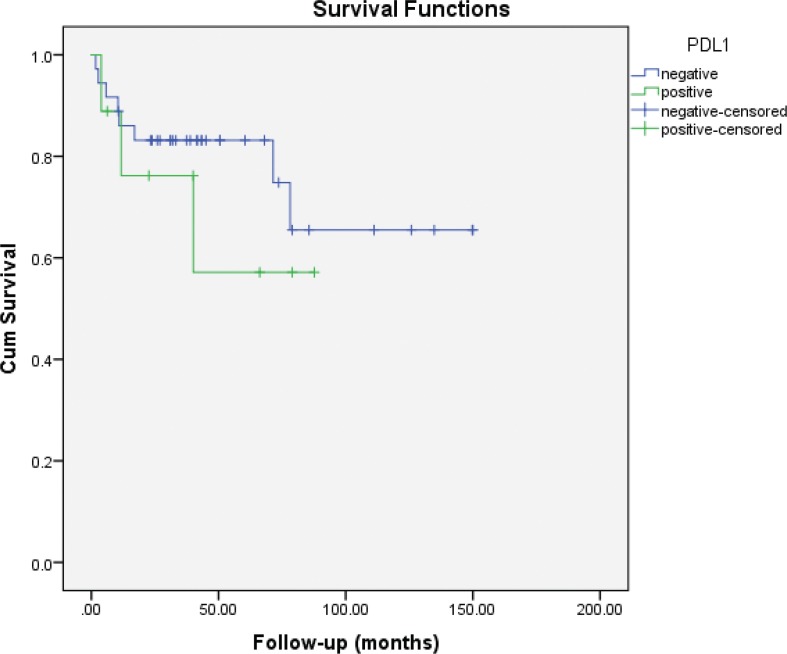
Kaplan–Meier plots of cancer-specific survival (CSS) according to PD-L1 expression status in non-ccRCC patients.

**Figure 3:**
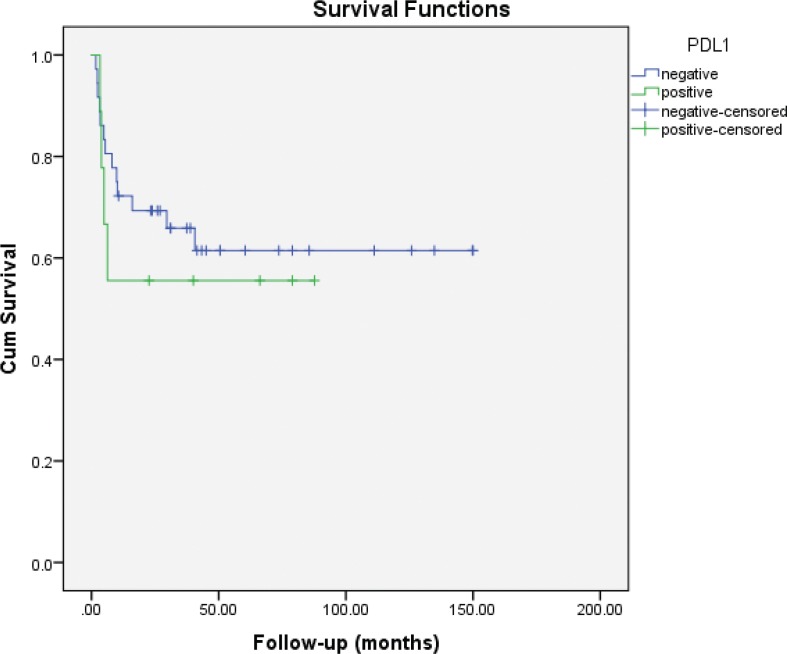
Kaplan–Meier plots of recurrence-free survival (RFS) according to PD-L1 expression status in non-ccRCC patients.

Median follow-up was 40 months (IQR: 22.9–72.5). Eleven (24.4%) patients had died of disease at the time of the analysis. Five-year CSS was 83 and 73% for PD-L1 negative and positive cases, respectively (log-rank P = 0.47) (Figure 2). Five-year RFS was 61.5 and 55.6% for PD-L1 negative and positive cases, respectively (log-rank P = 0.58) (Figure 3).

## Discussion

Immunomodulatory agents targeting the PD-1/PD-L1 axis have revolutionized treatment of several malignancies, including advanced RCC (10). Unfortunately, much of this progress has been limited to patients with ccRCC, partly due to the relative rarity of non-clear cell variants and the paucity of knowledge about the molecular and biologic drivers of disease. In this study, PD-L1 was expressed in a fifth of non-ccRCC cohort while being associated with adverse histopathologic features.

PD-1 is a member of the B7–CD28 family and serves as a cell surface inhibitory receptor on T cells (13, 14). Mechanisms of action of immune checkpoint inhibitors are nonspecific but generally lead to immune system activation (6, 15). Expression of PD-1 by tumor-infiltrating lymphocyte mononuclear cells (TIMC) and PD-L1 on tumor cells has been associated with poor outcomes in several tumor types (15–19). Thompson et al. were one of the first groups to demonstrate PD-L1 expression as a significant predictor of cancer progression and mortality in ccRCC (20, 21).

To date, only two studies evaluating PD-L1 expression in non-ccRCC have been published. Choueiri et al. assessed PD-L1 expression in 101 non-ccRCC patients including chromophobe (*n* = 36), papillary (*n* = 50), collecting duct (*n* = 5), and Xp.11.2 translocation (*n* = 10) variants (22). PD-L1 positivity on tumor cells (11 cases, 10.9%) was significantly associated with higher stage and Fuhrman grade, and a worse overall survival. Similarly, our study revealed that PD-L1 positivity was associated with higher Fuhrman grade and perineural invasion, with a trend towards worse oncological outcomes. Conversely, Abbas et al. found no significant correlation between PD-1/PD-L1 expression and oncological outcomes in 63 cases of papillary, chromophobe, and sarcomatoid RCC variants (23).

Because of the lack of available treatments, studies combining non-clear and clear cell carcinomas have focused on angiogenesis inhibitors (anti-VEGF) and other targeted therapies (mTOR inhibitors). A recent meta-analysis of 20 studies including 1244 non-ccRCC and 6300 ccRCC patients revealed that the objective response rate to targeted therapy was significantly lower in those with non-clear cell subtypes (9.2% vs 14.8%) (24). Progression-free survival and overall survival were also shorter in non-ccRCC (7.5 and 13.2 months) versus ccRCC patients (10.5 and 15.7 months). Further studies with non-ccRCC cohorts are needed to assess the immune checkpoint therapy for expanding therapeutic options for these patients.

We acknowledge the several limitations of this study, including inherent biases associated with its retrospective design, small sample size, and population heterogeneity. Although we correlated PD-L1 expression with oncologic outcomes, causality cannot be established. The limited number of cases and events underpowered our analysis. Nevertheless, this is a common problem due to the rarity of non-ccRCC and a main cause of poor accrual in clinical trials. Moreover, this study validates the findings of the only previous report showing a correlation between PD-L1 positivity and adverse histopathologic features in non-ccRCC.

## Conclusion

PD-L1 is expressed in non-ccRCC and is associated with a more aggressive tumor phenotype. There was also a trend toward worse oncological outcomes in patients with PD-L1-positive tumors. Expression of biomarkers such PD-L1 may help better risk-stratify non-ccRCC patients to guide treatment decisions and follow-up strategies. Further investigation of anti-PD-1 and anti-PD-L1 immune checkpoint inhibition in patients with non-ccRCC is warranted.

## Conflict of interest

The authors declare no potential conflicts of interest with respect to research, authorship and/or publication of this article.
